# Geographies of Dirty Water: Landscape-Scale Inequities in Coastal Access in Rhode Island

**DOI:** 10.3389/fmars.2021.760684

**Published:** 2022-01-27

**Authors:** Julia H. Twichell, Kate K. Mulvaney, Nathaniel H. Merrill, Justin J. Bousquin

**Affiliations:** 1 Atlantic Coastal Environmental Sciences Division, Center for Environmental Measurement and Modeling, Office of Research and Development, U.S. Environmental Protection Agency, Narragansett, RI, United States,; 2 Oak Ridge Associated Universities, Oak Ridge, TN, United States,; 3 Gulf Ecosystem Measurement and Modeling Division, Center for Environmental Measurement and Modeling, Office of Research and Development, U.S. Environmental Protection Agency, Gulf Breeze, FL, United States

**Keywords:** equity, distributional justice, coastal recreation, water quality, environmental justice, public access

## Abstract

Across the United States, development, gentrification, and water quality degradation have altered our access to the coasts, redistributing the benefits from those spaces. Building on prior coastal and green space access research, we examined different populations’ relative travel distances to all public coastal access and to public marine swimming beaches across the state of Rhode Island, by race, ethnicity, and socioeconomics. Next, we assessed relative travel distances to high quality public coastal amenities, i.e., sites with no history of water quality impairment. We used three state-level policy attributes to identify sites with the best water quality: Clean Water Act Section 303(d) impaired waters, shellfishing restrictions, and bacterial beach closure histories. Our analysis revealed statewide disparities in access to Rhode Island’s public coastal amenities. With robust socioeconomic and geographic controls, race and ethnicity remained strongly correlated to travel distance. Higher proportions of Black and Latinx populations in census block groups were associated with longer travel distances to public access, in particular to public coastal sites with better water quality and to public swimming beaches. This translates to added costs on each trip for areas with higher Black and Latinx populations.

## INTRODUCTION

Coastal areas are highly valued but are not always equitably accessible. Coastal and near-coastal residents and visitors receive a variety of physical, psychological, health, and social benefits (Ashbullby et al., 2013; White et al., 2014; Nutsford et al., 2016; de Bell et al., 2017). In addition, many people benefit from fishing and shellfishing for recreational purposes, for subsistence, and to support their livelihoods (Burger, 2002; Lauber et al., 2017). Several factors influence shoreline access. Time, cost, transportation, parking availability, and weather can pose barriers to coastal visitation (García and Baltodano, 2005; Ashbullby et al., 2013; Patrolia et al., 2017). People are more likely to visit salt and fresh waters that are closer to their residence (Giles-Corti and Donovan, 2002; Cox et al., 2006; Haeffner et al., 2017). Site quality such as litter, vandalism, and perceived water conditions impact visitor preferences (Cox et al., 2006; Lyon et al., 2018). Areas with working waterfronts, shoreline armoring, and privatization of coastal access may physically or logistically restrict certain types of recreational use or resource use (Griffith, 2000; Mongeau, 2004; García and Baltodano, 2005).

An individual’s resources, where they reside, and the quality of the natural amenities available may impact their access to the shoreline; thus, coastal access has potential environmental justice implications. The recurrent story from many environmental justice studies has been one where under-resourced communities are disproportionately exposed to a range of disamenities such as contaminated zones and sources of air, soil, and water pollution (e.g., Bullard, 2000; United Church of Christ, 2007; Mohai et al., 2009). The field has also expanded to consider disparities in access to amenities, such as green space (e.g., Boone et al., 2009; Zhang et al., 2011; Wen et al., 2013; Casey et al., 2017; Watkins and Gerrish, 2018); however, less is known about access to water amenities.

Spatial analysis through an environmental justice lens can help to elucidate the benefits available from water resources to different populations, but relatively few studies are available. Sanchez et al. (2014) found a weak relationship between census tracts with higher social vulnerability and the presence of freshwater streams with poor biological condition in a Michigan watershed. In Utah, higher-income white populations reported that they lived farther from urban freshwater canals and rivers but also reported more frequent use than non-white populations (Haeffner et al., 2017). Montgomery et al. (2015) found that Latinx areas and areas with reduced socioeconomic status were disproportionately farther from public beach access in Miami, Florida. Meanwhile, Kim et al. (2019) did not find a relationship between proximity to beaches and race/ethnicity in Detroit, Michigan.

This study undertakes a statewide geospatial analysis of coastal public access and community demographics in Rhode Island, United States and is also the first to incorporate measures of site quality, a key variable identified in similar park access studies (Rigolon, 2016). Specifically, we explored geographic patterns by race (white, Black, Asian, Indigenous American), ethnicity (Latinx), and several socioeconomic variables (e.g., income, home value, etc.). We build on methods from the work of Montgomery et al. (2015) and Kim et al. (2019) by expanding our analysis to all types of coastal public access points—including a focused analysis of public marine swimming beaches—and by examining access to coastal sites with the best water quality. We present a policy-oriented analytical approach focused on producing clear metrics to support efforts to improve racial equity around coastal resources.

## MATERIALS AND METHODS

This study examines access to all types of public coastal sites and to swimming beaches in conjunction with water quality for the state of Rhode Island, United States.

### Site Profile

**FIGURE 1 |:**
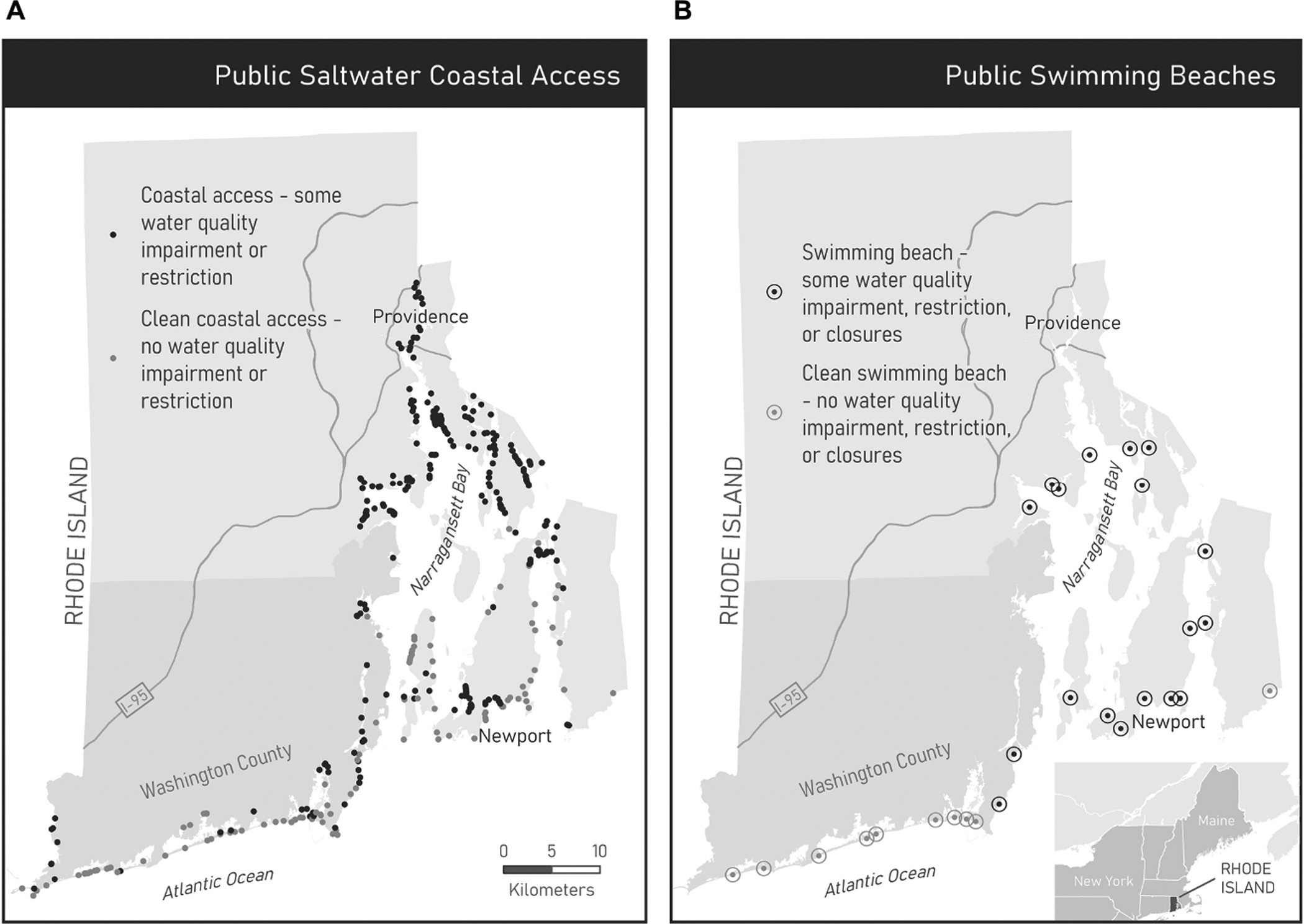
**(A)** Public saltwater coastal access points (*N* = 410). **(B)** Public marine swimming beaches (*N* = 28). Clean sites symbolized in light gray; all other sites symbolized in dark gray. Clean is defined as no history of state-assessed water quality-related impairments, restrictions, or beach closures (2012–2016, beaches only).

Rhode Island is nicknamed “The Ocean State” for its roughly 1300 km of saltwater shoreline (Figures 1A,B). In addition to an expanse of shoreline abutting the Atlantic Ocean, the state’s coastal geography is dominated by New England’s largest estuary, Narragansett Bay, and a set of coastal salt ponds. The saltwater coastline in Narragansett Bay extends in a variety of embayments in densely settled coastal areas and highly urbanized areas surrounding Providence, the state capital and primary population center located in the upper estuary. Water quality declines from the tidally flushed areas in the south to the terrestrially influenced upper saltwater reaches of Narragansett Bay (Narragansett Bay Estuary Program, 2017). Because of Rhode Island’s small size (roughly 65 by 80 km), all residents live relatively close to the coast and thus should have access to coastal recreation.

This paper analyzed 410 saltwater public access points published by the Rhode Island Coastal Resources and Management Council (Figure 1A). We excluded public access points not accessible by car (i.e., only accessible by ferry or private vessel). Coastal access points include rights-of-way, boat ramps, fishing sites, overlooks, conservation areas, parks, and beaches, all supporting diverse uses of the coast. A focused analysis of the 28 public marine swimming beaches included eight beaches operated and maintained by the state, eighteen operated and maintained by towns, and two privately operated public beaches (Figure 1B). Town beaches that do not allow for non-resident visitation were excluded because this study only considered sites that are publicly accessible to all state residents.

Demographics across Rhode Island follow strong geographic patterns. In 2016, the American Community Survey estimated that 74 percent of the state population identified as white alone (non-Latinx), higher than the national average of 61 percent (U.S. Census Bureau ACS 5-Year Rhode Island Data Profiles, 2012–2016). Most Latinx (14 percent), Black (7 percent), and Asian populations (3 percent) resided in the area around the City of Providence, with the latter more dispersed. Fewer than one percent of Rhode Island’s population identified as Indigenous American with the greatest proportion residing in the Providence area. The Narragansett Indian Tribal Reservation is located in the southern part of the state. Historically, the Narragansett tribal territory spanned Rhode Island.

### Coastal Proximity Indicators

This study applies and builds on the multiple regression techniques used by Montgomery et al. (2015) and Kim et al. (2019) to examine access to all types of public coastal sites and to swimming beaches in conjunction with water quality. Data were examined at the census block group scale, which is the smallest geographic unit for which the U.S. Census Bureau aggregates and publishes detailed demographic data. American Community Survey (ACS) 5-year estimates were used because of the increased statistical precision compared to 1-year estimates for census block group units. Residents are likely to travel outside of their census block group to access the shoreline; thus, we developed proximity indicators as opposed to the containerbased approaches typically used in environmental justice studies of green space accessibility (e.g., Wolch et al., 2005; Hughey et al., 2016). We constructed two sets of proximity indicators using travel distances between census block group centroids (U.S. Census Bureau ACS 5-Year Data Profiles, 2012–2016; *N* = 811) and coastal public access points (*N* = 410, beach subset = 28) (Table 1). Road network travel distances for all possible origindestination pairs were calculated using ESRI ArcGIS Online Connect Origins to Destinations (Esri, last accessed October 2019). The use of a distance-based approach in environmental justice analysis means that all “exposure” to each census block group—in this case exposure to environmental amenities—is considered and the variations in block group size and delineation do not determine the exposure (Banzhaf et al., 2019).

The first set of proximity indicators evaluated mean travel distance from each census block group centroid to the nearest public coastal access points (A) or public beach (C) (Table 1). For evaluation across all types of access sites (*N* = 410), the measure summarized travel distances to the ten closest sites. This was designed to reflect coastal visitation choices that may be related to proximity, quality, amenities, parking, type of access point, and congestion (e.g., Cox et al., 2006; Ashbullby et al., 2013; Haeffner et al., 2017). The cutoff of the ten nearest sites was consistent with human information processing theory, which suggests that human capacity to make judgments is limited to seven plus or minus two elements in a set (Zhang et al., 2011; Montgomery et al., 2015). In addition, because the public dataset may not account for coastal access through undocumented, locally known coastal access points, summarizing travel distance across ten sites better accounted for travel to other coastal access sites that might fall in between. The beach analysis (*N* = 28) examined travel distances to the nearest single public swimming beach, allowing exploration of the best public swimming option available to residents.

Rigolon’s (2016) review of 49 studies of urban park access found that low-income and non-white groups had consistently less access to higher quality parks; thus, the second set of proximity indicators (B and D) incorporated water quality characterization at each access point or beach (Table 1). Three state-level policy datasets were used to identify sites with impairments: Rhode Island Department of Environmental Management 2014 303(d) estuarine impaired water body listings (Rhode Island Department of Environmental Management, 2015), Rhode Island Department of Environmental Management 2017 shellfish harvest waters classification (Rhode Island Department of Environmental Management, 2017), shellfish harvest waters classification, and Rhode Island Department of Health 2012–2016 swimming beach bacteria closures. “Clean” water was thereby defined by this study as areas with no state-assessed water quality impairment, shellfishing restrictions, or recent history of closures (beaches only). These datasets come with inherent limitations for accurately representing water quality at a site; however, by combining multiple datasets we were better able to exclude any sites with evidence of poor water quality. As such, we excluded all public coastal access sites on waterbodies with any type of 303(d) impairment, based on the state’s 5-year rotating assessment of all estuarine water bodies for pathogens and toxins, as well as assessments of other water quality parameters such as nutrient and oxygen levels (Rhode Island Department of Environmental Management, 2015). We excluded coastal access sites within areas classified as restricted or prohibited for shellfish harvest, as those prohibitions are based on bacterial sampling to be protective of human health as a result of consuming shellfish. Finally, for the public beach analysis only, we excluded swimming beaches with any bacterial closures between 2012 and 2016. We examined a five-year record of beach closures because swimming beaches may develop a reputation for poor water quality due to the closure history. We identified 120 clean public access points and ten clean public swimming beaches (Figures 1A,B). The second set of proximity indicators evaluated mean travel distance to the ten closest, clean public coastal access sites (B) and mean travel distance to the nearest clean public swimming beach (D).

### Data Analysis

**FIGURE 2 |:**
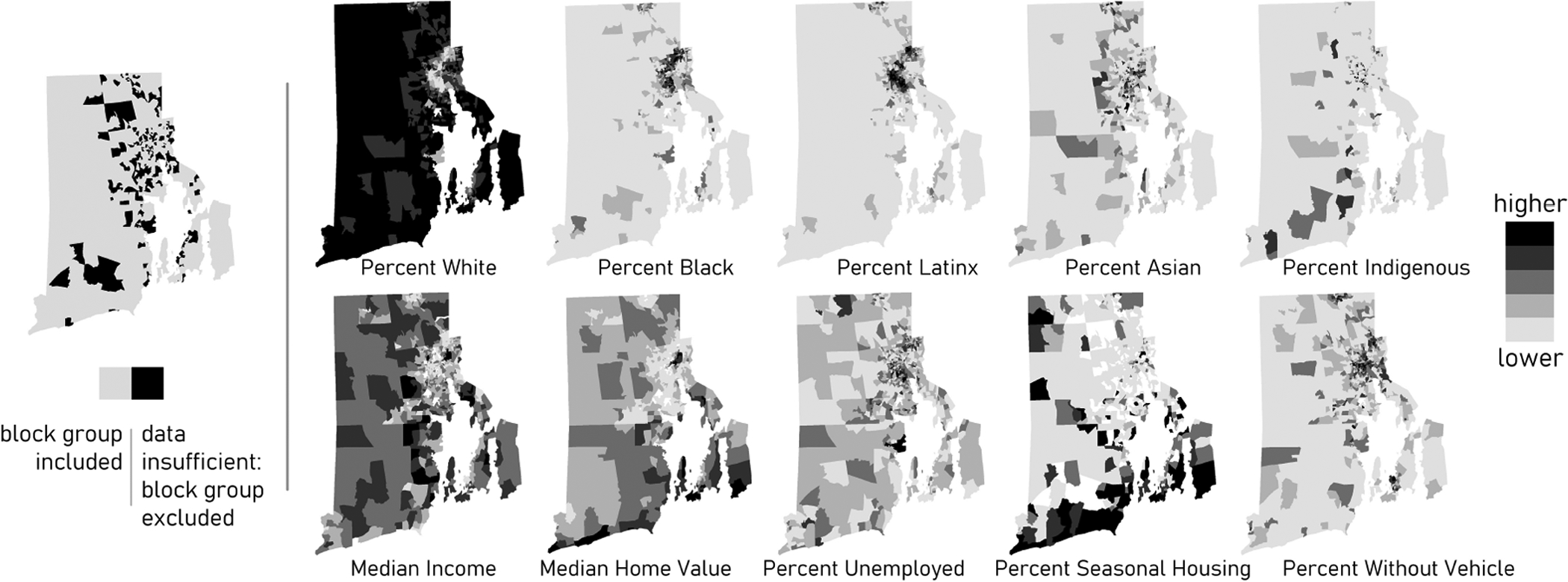
On the left, 206 census block groups (in black) were excluded from the regression analyses due to insufficient data for some of the demographic variables. On the right, choropleth maps of demographic variables, symbolized using five-class natural breaks to optimize the uniformity of values in each class [U.S. Census American Community Survey (ACS) 5-Year Data Profiles, 2012–2016]. Darker shades indicate higher values.

We sought to examine the relationship between census block group demographics and travel distances to the nearest coastal public access sites, the nearest beaches, as well as to the nearest clean sites. We specifically investigated the relationship with race and ethnicity while controlling for socioeconomics and the underlying spatial variation present in the study area using ordinary least squares regression. Controls were selected based on previous use in green space and water recreation accessibility literature and hypothesized relationships (Table 2). The socioeconomic factors selected have been variously used to describe degrees of disadvantage, economic resources, neighborhood economic insecurity, and/or instability (e.g., Wolch et al., 2005; Montgomery et al., 2015; Hughey et al., 2016; de Bell et al., 2017). Census data were converted to percentages to support comparison across block groups with different populations. Visualization in five-class choropleth maps aided understanding of the spatial patterns exhibited by different variables (Figure 2).

The location characteristics of each origin block group were controlled for *via* geographic factors including Euclidian distance to the coast, a dichotomous variable for urbanized areas, and a dichotomous variable for Washington County, which encompasses the southwestern section of the state. It comprises the majority of the coastal areas with the cleanest water quality along the coast and many of the state’s public swimming beaches. It is also the least diverse of the five counties in Rhode Island and has some of the state’s highest property values (Table 2).

Ordinary Least Squares (OLS) regression was used to analyze global patterns for the two sets of proximity indicators. For each of the four indicators (A, B, C, D), the five race/ethnicity variables were run separately because they sum to nearly 100 percent and are highly correlated, increasing redundancy and collinearity in the regressions (Montgomery et al., 2015). For each of these twenty regressions, a suite of five sequentially more specified regressions were run to parse out the relationships between controls, race/ethnicity variables, and the proximity indicators. All regressions used the same sets of variables. Some census block groups lacked reporting for several socioeconomic variables. Those lacking data were excluded, which reduced N to 605. This method was designed not to establish causality, but rather to test for the existence of relationships and control for the interconnections between race and ethnicity, socioeconomics, and the underlying spatial variation present in the study area.

Spatial data often exhibit spatial autocorrelation, or spatial clustering, where features tend to have more similarity with features that are closer compared to those that are farther away. Spatial autocorrelation causes issues in regression and can produce less precise models and inaccurate (overconfident) confidence intervals around coefficient estimates. We account for the spatial autocorrelation and inconsistent variance in the error terms (heteroskedasticity) present in all models *via* use of heteroskedasticity and autocorrelation consistent (HAC) estimators (also called Newey-West estimators) to produce corrected, robust standard errors (e.g., Zeileis, 2004; Anselin and Lozano-Gracia, 2008).

Finally, variation in round-trip costs were tabulated from the regression results to create policy-oriented metrics. The aim was to illustrate, more intuitively, the associations between race and ethnicity and travel distance from any census block group to the nearest and cleanest public coastal access sites. The additional distance represents a financial cost in terms of time and the associated costs of transport. Costs were calculated from the travel distance coefficients associated with the race and ethnicity variables (for illustrative purposes, marginal changes were based on a change of ten percent of the white, Black, or Latinx population for any given block group). These costs reflect the differences in the cost of gasoline and wear and tear for a round trip and the value of the amount of time spent traveling as it relates to the proportions of any race/ethnicity group for a given block group (Equation 1).

(1) $$\Delta tc=\frac{\beta *10*2}{\text{speed}}*\text{income}+\left(\beta *10*2\right)*\text{permile}$$

*Where*:

Δ*tc* – difference in round trip travel costs for a 10% increase in race/ethnicity group.

β – Coefficient from regression 5 for race/ethnicity group. The difference in distance for a 1% increase in the proportion of a race/ethnicity group.

*income* – 2016 median income for census block groups in Rhode Island ($28.99/h).

*speed* – mean vehicle speed of 35 km per hour.

*permile* – 2016 IRS mileage reimbursement rate of 0.332 dollars per kilometer.

How best to treat the opportunity cost of time in recreation demand models is unsettled, but common practice in the non-market economic valuation literature is to use a fraction of the wage rate. We did not seek to create results appropriate for recreation demand modeling or non-market valuation applications and, for simplicity, we used the whole hourly wage rate in this analysis. Furthermore, we chose to use a mean wage rate for the region, treating every race/ethnicity group’s value of time equally, unlike some travel-cost calculations that might vary this based on the respondent or group’s income (Lupi et al., 2020).

## RESULTS

Statewide summary statistics for all variables are presented in Table 2. Washington County statistics are provided as a comparison.

For each proximity indicator (A, B, C, D), a suite of five increasingly specified regressions were run to understand the relationships between controls, race/ethnicity variables, and the proximity indicators. As shown in the representative example in Table 3, adding levels of specification to the regression controls for covariates beyond race and income that might also be related to travel distance to coastal resources. The most highly specified regression (Regression 5, Table 3) indicates that the percent of the population identifying as non-Latinx white remained a significant factor when controlling for other variables that, together, accounted for 77 percent of the variance in travel distance to the nearest beach. As this pattern was consistent for all sets of regressions, this paper discusses only Regression 5 results (i.e., the most highly specified regression).

We generated a regression using HAC robust standard errors and corrected p values for all Regression 5 analyses (Table 3). A variable with a negative coefficient indicates a shorter travel distance as that variable increases, after controlling for the other factors. For example, Regression 5 indicates a negative relationship between the non-Latinx white population and travel distance to the nearest public beach that persists after controls. In the interest of producing policy-oriented metrics, one way to illustrate this is that after accounting for socioeconomic and travel-related factors known to influence travel distance, the model suggests that a ten percent increase in the non-Latinx white block group population was associated with being 0.93 km closer, by road, to the nearest public beach.

### Public Coastal Access

We observed disparities in mean travel distance to the nearest ten public coastal access sites between some populations (proximity indicator A regressions; Table 4). Results for the three predominant race and ethnicity groups in Rhode Island are presented below (non-Latinx white, Black, Latinx). The results for Indigenous American and Asian populations are available in Supplementary Appendix A, but were generally not associated with statistically significant differences in travel distance. It is possible that public coastal access opportunities vary for these groups, but this analysis is limited in its detection power because the populations in Rhode Island are small (Rhode Island’s population is three percent Asian and less than one percent Indigenous American).

Overall, the mean travel distance from any census block group in Rhode Island to the ten nearest public access points was 8.6 km. After controlling for socioeconomic and travel-related factors, a ten percent increase in the non-Latinx white block group population was associated with being 0.29 km closer, by road, to the nearest ten public access points (*p <* 0.001). An increase in the Black population was not associated with a statistically significant change in travel distance. A ten percent increase in the Latinx population was associated with a 0.45-km longer travel distance from access points (*p <* 0.001).

Euclidean distance to the coast and urbanization were associated with a statistically significant change in travel distance to the ten nearest public coastal access sites. Those farther from the nearest coastline had higher mean travel times to the nearest access points. Populations in urbanized areas were associated with being closer to their nearest ten access points, reflecting the many access points available in the upper Narragansett Bay. Regressions for proximity indicator A accounted for 95 percent of the variance.

Table 4 also shows significant disparity in mean travel distance patterns to the nearest ten clean public access points in kilometers (regressions for proximity indicator B). Mean travel distance from any census block group in Rhode Island to the nearest ten clean public access points was 34.8 km. After the application of controls, a ten percent increase in the non-Latinx white block group population was associated with a 1.07-km shorter travel distance, by car, to the nearest ten clean public access points (*p <* 0.01). Ten percent increases in Black or Latinx populations were associated with being 1.78 (*p <* 0.01) and 0.90 (*p <* 0.05) kilometers farther, respectively.

More than half of the clean public access points in the state are in Washington County and located primarily outside of densely developed areas (Table 2). Focusing the analysis solely on clean sites removed nearly all access points in the upper reaches of Narragansett Bay and in protected embayments where tidal flushing is reduced, and terrestrial influences of urbanization are greater. Thus, block groups in Washington County with higher median home value and higher seasonal housing tend to be closer to the nearest clean access points and urbanized areas and areas with higher population density tend to be farther, as reflected in the regression results. Conversely, areas with higher median income were slightly farther on average from clean access points, controlling for other factors. Regressions for proximity indicator B accounted for 72–73 percent of the variance.

### Public Beach Access

Disparities in travel distance to the nearest public swimming beach were observed among some populations (Regressions for proximity indicator C; Table 5). Again, results for the three predominant race and ethnicity groups are presented below (non-Latinx white, Black, Latinx). Results for Indigenous American and Asian populations are available in Supplementary Appendix A; no statistically significant differences in travel distance were observed.

Mean travel distance from any census block group in Rhode Island to the nearest public swimming beach was 16.8 km. After controlling for socioeconomic and travel-related factors, a ten percent increase in the non-Latinx white block group population was associated with a 0.93-km shorter travel distance, by car, to the nearest beach (*p <* 0.001). Ten percent increases in Black or Latinx populations were associated with being 1.81 (*p <* 0.001) and 0.68 (*p <* 0.1) kilometers farther, respectively. Regressions for proximity indicator C indicate that as Euclidean distance to the coast and population increase, travel distance to public beaches also increases. An increase in unemployment and seasonal housing was associated with shorter mean travel distances to the nearest beach. Regressions for proximity indicator C accounted for 75–76 percent of the variance.

Analysis indicated that travel distance to the nearest clean public beach also varied by race and/or ethnicity (Table 5). Mean travel distance from any census block group to the nearest clean beach was 50.6 km. Following the application of controls, a ten percent increase in the non-Latinx white block group population was associated with a 1.03-km shorter travel distance, by car, to the nearest clean beach (*p <* 0.01). A ten percent increase in the Black population was associated with a 2.05-km longer travel distance (*p <* 0.001). A ten percent increase in the Latinx population was associated with a 0.64-km longer travel distance (*p <* 0.10).

The relationships between controls and the clean beach proximity indicator (D) were similar to those observed for proximity indicator C. However, likely because nine out of the ten clean beaches were located along the south coast of Washington County, urbanized areas are significantly farther from clean beaches. In addition, median income was positively correlated to travel distance to clean beaches. Regressions for proximity indicator D accounted for 75–76 percent of the variance.

### Travel Costs

**FIGURE 3 |:**
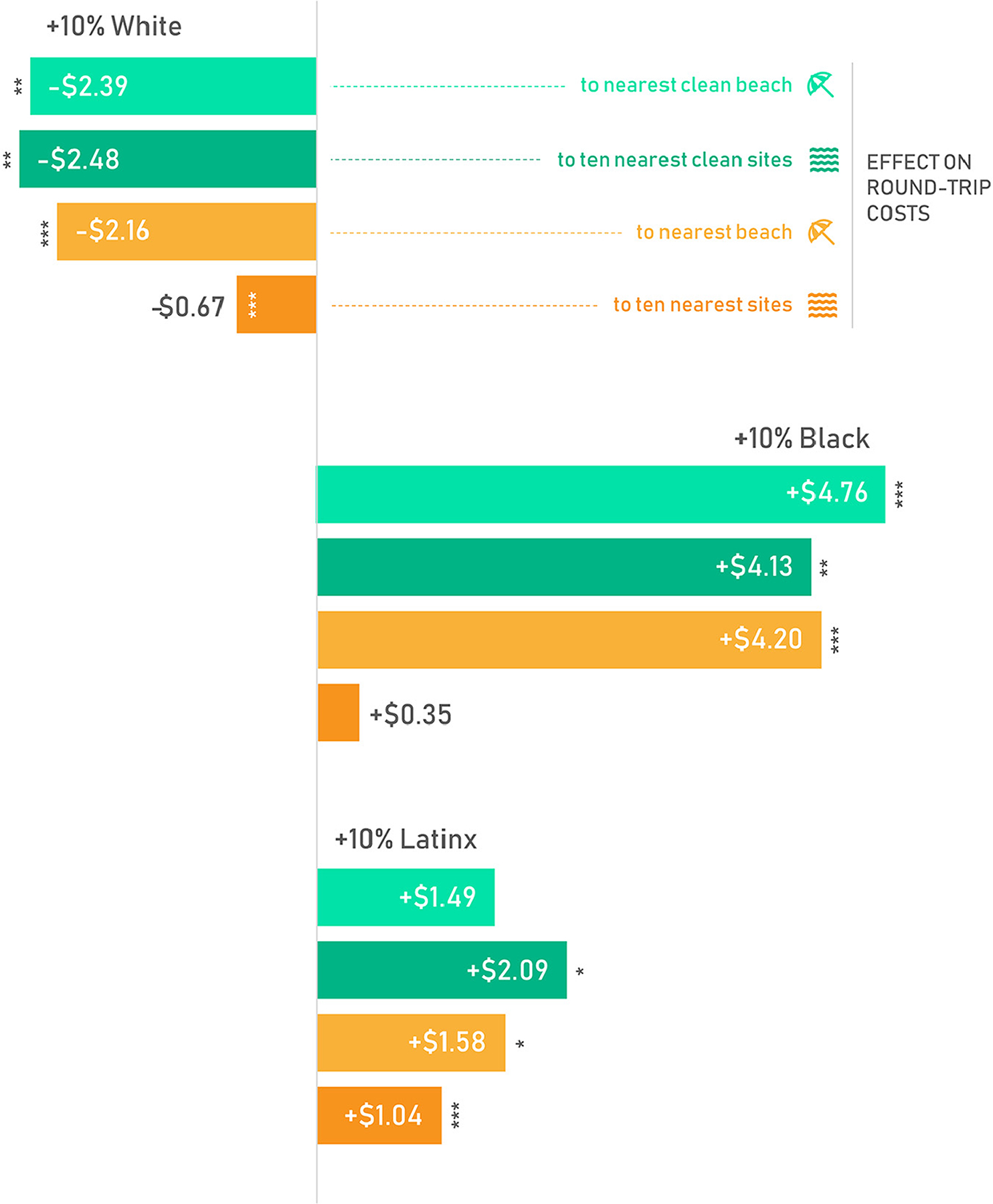
Change in round-trip travel cost by race and/or ethnicity to public access points and public beaches. Differences in travel costs are calculated from travel distance coefficients. These can be interpreted as change in cost for travel from any block group that would be associated with a ten percent increase in the white, Black, or Latinx population. Stars indicate the level of significance for the change in travel distances, from regressions in Tables 4, 5 (*p < 0.05; **p < 0.01; ***p < 0.001). Travel costs in USD($) are based on the 2016 IRS mileage reimbursement rate of 33.2cents/km, a mean vehicle speed of 35km/h, the 2016 median income for RI of $60,201 ($28.99/h), and the coefficient from regression 5 for the race/ethnicity group multiplied by two. Parking/fees not included.

Variations in travel cost per round trip were tabulated from the race and ethnicity variable regression 5 coefficients to illustrate the relationships between race and ethnicity and travel distance. These coefficients are associated with the p values presented in Tables 4, 5 (Figure 3). To create policy-oriented metrics, we tabulated incremental variations in travel cost that are associated with a ten percent increase in the white, Black, or Latinx populations for a block group after controlling for socioeconomic and travel-related factors.

Travel cost tabulations indicate that a 10% increase in a block group’s Black population was associated with an increase, on average, of $5 more per round trip. Similarly, each ten percent increase in a block group’s Latinx population was associated with an added cost of up to $2 per trip. By contrast, each ten percent increase in a block group’s non-Latinx white population was associated with up to a $2.50 savings per trip. The disparity in cost per trip was larger for high quality public coastal sites and public swimming beaches than the variations in travel cost were to all types of coastal public access (Figure 3).

Based on a 2012 ocean recreation survey (Kosaka and Steinback, 2012), New England residents make on average 24 ocean recreation visits per 12-month year. As such, costs per trip add up to a non-trivial impact per year to Black and Latinx populations that typically have lessor means to accommodate the additional cost. Meanwhile, non-Latinx white populations with typically greater means to afford the costs of recreation see greater savings. Furthermore, travel cost alone does not account for the value of a person’s leisure time, and the relative scarcity of that leisure time for those who work more hours for lower pay.

## DISCUSSION

This research explored statewide patterns in access to public coastal areas across different demographics. Our findings provide clear metrics that indicate unequal travel distances and costs to visit public coastal amenities, especially beaches and coastal sites with the best water quality, for different populations in the state of Rhode Island. Inequity along the divides of race and ethnicity occurs in the presence of economic inequalities and other factors (Downey, 1998). We controlled for a variety of factors besides race and ethnicity and accounted for between 75 and 95 percent of the variance across the state. These factors included socioeconomic attributes as well as spatial setting attributes such as distance to any coast. Despite these robust controls, correlations between travel distance to coastal amenities and race/ethnicity persist strongly, revealing inequity. Results such as these highlight an opportunity to counteract disparities by reducing travel barriers to high quality coastal areas and through clean-up efforts focused on public coastal access used by or in closer proximity to under-resourced populations.

Pulido (2000) illustrates that environmental justice issues may derive from multidimensional forms of racism. These include both explicit discriminatory actions (e.g., the siting of environmental hazards) and the underlying, often invisible, structural processes that have benefitted white populations in the United States, such as white flight and suburbanization, housing opportunity and affordability, and white privilege (Wilson, 2006; Rothenberg, 2008; Mohai et al., 2009). Across the United States, distinct geographies of race and ethnicity in urban and suburban areas have been etched in the landscape by a legacy of greater opportunities historically afforded to white and/or higher income populations. These opportunities have enabled relocation away from disamenities and toward more desirable, suburbanized, clean areas with better amenities and resources (Pulido, 2000; Kuswa, 2002).

Reflecting national trends, Rhode Island’s white population shifted away from the heavily urbanized areas around Providence to suburbanized areas during the post-war era and late-twentieth century (Jerzyk, 2009). This included shifts to the prime areas along the Atlantic Coast, which have attracted wealthier residents and seen property values rise over the years. Coastal waters provide leisure spaces that are now integral to the Rhode Island identity. Although Narragansett Bay has recovered significantly due to a variety of restoration and management efforts, water quality challenges remain in the northern reaches of the bay and some smaller embayments where highly developed areas face greater anthropogenic pressures (Narragansett Bay Estuary Program, 2017; Figures 1A,B). The landscape-scale view taken in this study suggests that the coincident geographies of poorer water quality and urbanized areas with larger non-white populations could impact the dynamics around the use of clean public coastal access. This is simply because greater travel distances to visit public beaches and public coastal sites with better water quality result in greater recreation costs for groups that live in those areas.

Several additional factors above and beyond travel distance are likely to mean that non-white populations may be underrepresented in coastal recreational spaces. In Rhode Island, mean travel distance to clean coastal sites and beaches ranges from 30 to 50 km. The lack of resources such as access to a car, gas money, or time away from childcare and the burden of working several jobs may make longer travel distances to the coast prohibitive. Parking at coastal sites is often limited and many prime beaches in the state require payment to park (non-residents can pay up to 30USD per weekend day). Lower participation in outdoor pursuits and higher drowning risks have been linked to historically discriminatory practices, racialized spaces, and lack of access (Wiltse, 2014; Fernandez et al., 2015; Lee and Scott, 2016). Additional research will be needed to understand how accessibility in those dimensions relates to visitation at different types of sites for different demographics.

The dynamics around the accessibility and use of coastal spaces have social consequences. Reduced access to green and blue space has been associated with a number of health risks. Positive leisure habits, such as exercise and socialization, and the associated mental and physical health benefits have been linked with access to green and blue space (e.g., Völker and Kistemann, 2011; White et al., 2014; Lee and Scott, 2016; de Bell et al., 2017; Theriault and Mowatt, 2018). Many families may not have easy respite from heat stress in urban areas. A number of families living in urban areas in Rhode Island fish and recreate, and children play, in the waterways near to their homes that routinely test positive for fecal contamination or that have been determined to be impacted by a variety of other environmental stressors, including heavy metals (Woonasquatucket River Watershed Association, personal communication, June 2019). Under-resourced populations may thus be at a greater health risk for mental and physical ailments, heat stress, and bacterial infection. This study indicates one pathway to address this through more equitable access to clean public coastal sites.

Water quality policy and restoration efforts have been shown to vary spatially in relation to the demographics of residents in other areas (Dernoga et al., 2015; Stanford et al., 2018). The patterns observed in this study can be useful for preparing decision makers to better serve their communities and account for inequity that manifests at a regional scale (Figure 3). Considerable efforts have been made thus far to increase and maintain public coastal access in Rhode Island and to restore coastal water quality. Augmenting public coastal access in urban areas could increase opportunities for coastal activities particularly if coincident with water and air pollution management and improvements to physical site quality. Consideration for the impact to and needs of local communities will be important to enrich the health of all communities and avoid displacement *via* gentrification (Essoka, 2010).

Underserved communities could also substantially benefit from the reduction of travel barriers to high quality coastal areas. Currently, inexpensive bussing is available from urbanized areas to two Rhode Island beaches on heat advisory days. This is an example of a policy-based solution that addresses travel barriers rather than water quality. Improving public transportation, fees, and parking could increase access to high-quality coastal sites and provide more options for all state residents to benefit from the shorelines.

### Areas for Future Research

This study’s results are aggregated to census block groups. Individual experiences may vary from the general population in a block group. The method uses travel distance from each block group to assess potential access (access opportunity) by different populations, given that greater use of coastal areas has been linked to residential proximity (Cox et al., 2006). This study does not describe the actual use of coastal areas. Future research identifying the demographic characteristics of actual coastal resource users at different types of public coastal sites will be important and complementary to this study.

This analysis considers all types of coastal public access points, which support a variety of recreational uses, as one aggregate group. We do not distinguish between the use of different types of coastal areas beyond the separate analysis of public marine bathing beaches. In addition, accessibility to different sites may differ substantially depending on site amenities, entrance fees, activity permits, and the availability of public transportation. Alternative recreational options may also impact site use. This analysis focused on saltwater public coastal access and did not include undocumented, locally known coastal access points, although the methods were designed to account for those options to some extent. It excluded freshwater alternatives, water and splash parks, and swimming pools that may be used for similar activities. There are a large number of freshwater access points and beaches in Rhode Island that are used heavily during summer months for water-related recreation. Other outdoor recreation areas such as public parks and trails could also draw activity away from coastal sites. Future consideration of how site type, alternatives, accessibility, amenities, and site quality (other than water quality) impact the use of coastal sites by different demographics will be needed.

The socioeconomic impacts of water quality are challenging to isolate due to the complicated spatial nature of upstream pressures and downstream impacts. Further research on demographic shifts around water quality over time (e.g., Youth et al., 2016) could provide greater understanding of the role that geography plays in enforcing structural classism and environmental racism around the coastline. This paper does not investigate causal links to these processes; however, capturing and reporting these patterns can inform efforts to reduce the inequities embedded in the landscape. Examining temporal patterns might further inform management of point and nonpoint source pollution, climate change, and sea level rise by increasing our understanding of who may benefit or lose out as coastal areas continue to change, and efforts are taken to mitigate or adapt.

## CONCLUSION

This statewide analysis of public coastal access in Rhode Island revealed landscape-scale inequities in the travel distances and costs for visiting public coastal amenities that were associated with race and ethnicity. Black and Latinx populations had longer travel distances to public coastal sites with good water quality, to public swimming beaches, and to the highest quality public beaches in the state. This translates to added costs on each coastal recreation trip for census block groups with higher Black and Latinx populations. The disparity was larger with regards to access to high quality public coastal sites and public swimming beaches, which reflected the dearth of high-quality public access in urban areas. Inequities around public coastal access may potentially diminish recreationrelated benefits and health outcomes for Black and Latinx populations while also costing those populations more to recreate. The results of this study equip policymakers and managers with clear metrics that can bolster their efforts to address these disparities in the state of Rhode Island. This analytical approach targeted at producing policy-oriented metrics is broadly relevant for other areas grappling with similar patterns of development, gentrification, and water quality degradation.

## Supplementary Material

Supplement1

## Figures and Tables

**TABLE 1 |:** Proximity indicators.

Set	Public coastal access (*N* = 410) proximity indicators	Public beach (*N* = 28) proximity indicators
1	A: Mean road network travel distance (TD) to nearest 10 coastal access points (km)	C: Mean TD to nearest beach (km)
2	B: Mean TD to nearest 10 clean coastal access points (km)	D: Mean TD to nearest clean beach (km)

**TABLE 2 |:** Summary statistics (2016) for all variables in Rhode Island and Washington County, RI.

Variable	Rhode Island	Washington County
**Proximity indicators**		
A: Mean TD to nearest 10 coastal access points (km)	8.6	7.4
B: Mean TD to nearest 10 clean coastal access points (km)	34.8	10.6
C: Mean TD distance to nearest beach (km)	16.8	9.5
D: Mean TD to nearest clean beach (km)	50.6	14.3
**Variables**		
Percent non-Latinx white	74.0	91.3
Percent Latinx	14.1	2.9
Percent Black or African American	6.5	1.6
Percent Asian	3.3	1.9
Percent Indigenous American	0.5	0.9
Median income (USD)	63,083	76,101
Median home value (USD)	250,957	360,314
Percent unemployment	34.7	35.4
Percent seasonal housing units	31.4	75.1
Percent with no vehicle	9.9	5.7
Euclidean distance to coast (km)	4.4	4.6
Urbanized area	(percent) 77.1	(percent) 32.6
Population/acre	1.5	0.1
**Coastal Access Descriptive Statistics**		
Clean* public access points (count / percent)	120/29.3	58/55.8
Total public access points	410	104
Clean* beaches (count / percent	10/35.7	9/81.8
Total beaches	28	11

Variables represent census block group level characteristics.

* No history of water quality related restrictions or impairments.

**TABLE 3 |:** Set of regressions showing relationships of controls with proximity indicator C, mean travel distance to nearest public beach (km).

Relationships with proximity indicator “C”: Mean travel distance (km) to the nearest beach					
	OLS				HAC robust SE
Variable	1	2	3	4	5
Percent white (non-Latinx)	−0.041**	−0.019	−0.132***	−0.095***	−0.093 (0.024)***
Median income (per 100K)		3.915	1.369	2.246	1.479 (1.821)
Median home value (per 100K)		−2.757***	−0.415	−0.273	−0.136 (0.435)
Percent unemployment		−28.342*	−23.043***	−22.651***	−21.277 (6.474)**
Percent seasonal housing units		−6.130***	−5.001***	−3.807***	−3.117 (1.217)*
Percent with no vehicle			2.400	0.616	0.002 (3.270)
Euclidean distance to coast (km)			1.553***	1.633***	1.617 (0.077)***
Urbanized area (Yes = 1, No = 0)				2.117**	1.537 (0.810)
Population/square acre				0.163***	0.150 (0.043)***
Washington county (Yes = 1, No = 0)					−3.246 (2.028)
Constant	19.888***	25.315***	21.687***	14.541***	15.376 (2.536)***
Observations	806	605	605	605	605
R^2^	0.010	0.152	0.746	0.760	0.767
Adjusted R^2^	0.009	0.145	0.743	0.756	0.763
Residual Std. Error	11.156 (df = 804)	10.613 (df = 599)	5.814 (df = 597)	5.666 (df = 595)	5.591 (df = 594)
F Statistic	8.270** (df = 1; 804)	21.479*** (df = 5; 599)	250.944*** (df = 7; 597)	209.301*** (df = 9; 595)	195.140*** (df = 10; 594)

* p < 0.05;

** p < 0.01;

*** p < 0.001.

Associations of added controls are shown by significance levels and on the overall regression by adjusted R^2^ and F statistics. The Regression 5 coefficients with p values based on HAC robust standard errors (shown in parentheses in the last column) are presented in Tables 4, 5.

**TABLE 4 |:** Linear regression results for proximity indicator (A) mean travel distance (TD) in kilometers to the nearest ten public access points and (B) mean TD to the nearest ten clean public access points, each run separately for race/ethnicity variables percent non-Latinx white (W), percent Black (B), and percent Latinx (L).

Travel distance (TD) to coastal public access						
	Relationship with travel distance (km)					
	Proximity indicator “A”: Mean TD to 10 nearest access points *Mean TD = 8.6km*			Proximity indicator “B” Mean TD to 10 nearest clean access points *Mean TD = 34.8km*		
Variable	A-W	A-B	A-L	B-W	B-B	B-L
Percent white (non-Latinx) (W)	−0.029***			−0.107**		
Percent Black (B)		0.015			0.178**	
Percent Latinx (L)			0.045***			0.090*
Median income (per 100K)	0.373	0.269	0.360	5.941*	5.853*	5.687*
Median home value (per 100K)	−0.176	−0.253*	−.155	−2.539*	−714*	−2.647*
Percent unemployment	−4.032	−1.508	−4.138	−16.658*	−13.680	−11.430
Percent seasonal housing units	−0.129	−0.131	−0.176	−7.793**	−7.772**	−7.898**
Percent with no vehicle	−2.119	−1.036	−1.974	−0.668	1.795	1.714
Euclidean distance to coast (km)	1.283***	1.271***	1.283***	1.576***	1.549***	1.552***
Urbanized area (Yes = 1, No = 0)	−1.113***	−1.142***	−1.020**	3.022	2.963	3.150
Population/square acre	−0.004	0.031	−0.008	0.176*	0.262***	0.235**
Washington county (Yes = 1, No = 0)	−1.365	−1.430	−1.408	−16.566***	−16.702***	−16.780***
Constant	6.804***	4.306***	3.934***	38.475***	28.832***	28.729***
Observations	605	605	605	605	605	605
R^2^	0.950	0.947	0.951	0.731	0.726	0.724
Adjusted R^2^	0.949	0.946	0.950	0.726	0.721	0.720
Residual Std. Error (df = 594)	1.846	1.905	1.821	8.835	8.911	8.938
F Statistic (df = 10; 594)	1,126.1***	1,053.6***	1,158.5***	161.2***	157.5***	156.2***
Moran’s I	0.339	0.394	0.313	0.363	0.357	0.385
z-score	42.5***	49.5***	39.3***	45.6***	44.8***	48.4***

* p < 0.05;

** p < 0.01;

*** p < 0.001.

Clean is defined as no history of water quality related restrictions or impairments. Regression coefficients (km) with associated p values generated from HAC robust standard errors.

**TABLE 5 |:** Linear regression results for proximity indicator (C) mean travel distance (TD) in kilometers to the nearest public swimming beach and (D) mean TD to the nearest clean beach, each run separately for race/ethnicity variables percent non-Latinx white (W), percent Black (B), and percent Latinx (L).

Travel distance (TD) to the beach						
	Relationship with travel distance (km)					
	Proximity indicator “C”: Mean TD to nearest beach *Mean TD = 16.8km*			Proximity indicator “D”: Mean TD to nearest clean beach *Mean TD = 50.6km*		
Variable	C-W	C-B	C-L	D-W	D-B	D-L
Percent white (non-Latinx) (W)	−0.093***			−0.103**		
Percent Black (B)		0.181***			0.205***	
Percent Latinx (L)			0.068*			0.064
Median income (per 100K)	1.479	1.466	1.233	6.628*	6.622*	6.318*
Median home value (per 100K)	−0.136	−0.262	−0.252	−1.498*	−1.637*	−1.659*
Percent unemployment	−21.277**	−20.091**	−16.034*	−25.387**	−24.223**	−18.593*
Percent seasonal housing units	−3.117*	−3.092*	−3.198*	−6.879***	−6.850***	−6.958***
Percent with no vehicle	0.002	1.796	2.298	5.877	7.840	8.751
Euclidean distance to coast (km)	1.617***	1.597***	1.593***	1.353***	1.332***	1.323***
Urbanized area (Yes = 1, No = 0)	1.537	1.496	1.620	4.952*	4.908*	5.009*
Population/square acre	0.150***	0.214***	0.210***	0.214**	0.284***	0.294***
Washington county (Yes = 1, No = 0)	−3.246	−3.340	−3.437	−30.873***	−30.976***	−31.097***
Constant	15.376***	6.937**	7.061**	51.163***	41.740***	42.014***
Observations	605	605	605	605	605	605
R^2^	0.767	0.763	0.755	0.763	0.761	0.757
Adjusted R^2^	0.763	0.759	0.751	0.759	0.757	0.753
Residual Std. Error (df = 594)	5.591	5.636	5.731	9.376	9.406	9.496
F Statistic (df = 10; 594)	195.1***	191.1***	182.8***	191.2***	189.6***	184.9***
Moran’s I	0.564	0.517	0.588	0.332	0.306	0.347
z-score	70.6***	64.9***	73.7***	41.7***	38.5***	43.6***

* p < 0.05;

** p < 0.01;

*** p < 0.001.

Clean is defined as no history of water quality related restrictions or impairments. Regression coefficients (km) with associated p values generated from HAC robust standard errors.

## Data Availability

The datasets analyzed in this study can be found at https://doi.org/10.5281/zenodo.5197235.
