# Supplementary material for: Geographies of Dirty Water: Landscape-Scale Inequities in Coastal Access in Rhode Island
Source: Front Mar Sci. Author manuscript; Available in PMC 2023 Jan 27. (PMC8903087; doi:10.3389/fmars.2021.760684)
Supplement: Supplement1 [file NIHMS1785270-supplement-Supplement1.docx]

**Appendix A.** Linear regression results for Asian and Indigenous American populations. Regression results show the effects on mean Travel Distance (TD) in kilometers to the to the nearest ten public access points, the nearest ten clean public access points, the nearest public swimming beach, and the nearest clean beach. Clean is defined as no history of water quality related restrictions or impairments. Regression coefficients (in km) are shown with confidence levels generated from HAC robust standard errors.

| **Travel distance (TD) to coastal public access sites and to the beach (km)** | | | | | | | | | |
| --- | --- | --- | --- | --- | --- | --- | --- | --- | --- |
|  | Effect on travel distance (in km) | | | | | | | | |
|  | Response variable “A”: Mean TD to 10 nearest sites Mean TD = 8.6km | | | Response variable “B”: Mean TD to 10 clean sites Mean TD = 34.8km | | Response variable “C”: Mean TD to nearest beach Mean TD = 16.8km | | Response variable “D”: Mean TD to clean beach Mean TD = 50.6km | |
| Explanatory Variable | A-A | | A-IA | B-A | B-IA | C-A | C-IA | D-A | D-IA |
| Percent Asian | 0.017 |  | | 0.149 |  | 0.031 |  | 0.091 |  |
| Percent Indigenous American |  | 0.099** | |  | 0.020 |  | 0.098 |  | 0.049 |
| Median Income (per 100K) | 0.234 | 0.240 | | 5.439* | 5.435 | 1.042 | 1.048 | 6.143* | 6.143* |
| Median Home Value (per 100K) | -0.273* | -0.264* | | -2.927** | -2.870** | -0.433 | -0.418 | -1.851* | -1.815* |
| Percent Unemployment | -0.628 | -1.676 | | -3.677 | -4.812 | -10.693 | -11.810 | -13.228 | -14.275 |
| Percent Seasonal Housing Units | -0.125 | -0.150 | | -7.724** | -7.818** | -3.117* | -3.151* | -6.845*** | -6.908*** |
| Percent with No Vehicle | -0.882 | -0.829 | | 3.675 | 3.963 | 3.934 | 4.015 | 10.154* | 10.339* |
| Euclidean Distance to Coast (km) | 1.268*** | 1.270*** | | 1.517** | 1.525*** | 1.571*** | 1.573*** | 1.299*** | 1.304*** |
| Urbanized Area (Yes=1, No=0) | -1.170*** | -1.157*** | | 2.691 | 2.894 | 1.385* | 1.419* | 4.705 | 4.826 |
| Population/square acre | 0.035 | 0.034 | | 0.309*** | 0.324*** | 0.275*** | 0.276*** | 0.347*** | -0.355*** |
| Washington County (Yes=1, No=0) | -1.430 | -1.520 | | -16.737*** | -16.865*** | -3.466 | -3.565 | -31.077*** | -31.183*** |
| Constant | 4.363*** | 4.398*** | | 29.524*** | 29.610*** | 7.705*** | 7.748*** | 42.580*** | 42.642*** |
| Observations | 605 | 605 | | 605 | 605 | 605 | 605 | 605 | 605 |
| R^2^ | 0.947 | 0.947 | | 0.722 | 0.720 | 0.749 | 0.749 | 0.756 | 0.755 |
| Adjusted R^2^ | 0.946 | 0.946 | | 0.718 | 0.715 | 0.745 | 0.745 | 0.752 | 0.750 |
| Residual Std. Error (df = 594) | 1.907 | 1.903 | | 8.974 | 9.010 | 5.793 | 5.794 | 9.517 | 9.529 |
| F Statistic (df = 10; 594) | 1,052.1*** | 1.056.4*** | | 154.4*** | 152.7*** | 177.7*** | 177.6*** | 183.8*** | 183.2*** |
| Moran’s I | 0.389 | 0.387 | | 0.386 | 0.384 | 0.580 | 0.575 | 0.347 | 0.340 |
| z-score | 48.9*** | 48.6*** | | 48.4*** | 48.2*** | 72.7*** | 72.1*** | 43.6*** | 42.7*** |

*p<0.05 **p<0.01 ***p<0.001
